# Supplementary figures and images for: Thermal Stability of Highly Filled Cellulosic Biocomposites Based on Ethylene–Vinyl Acetate Copolymer
Source: Polymers (Basel). 2024 Jul 24;16(15):2103. doi: 10.3390/polym16152103 (PMC11314597; doi:10.3390/polym16152103)

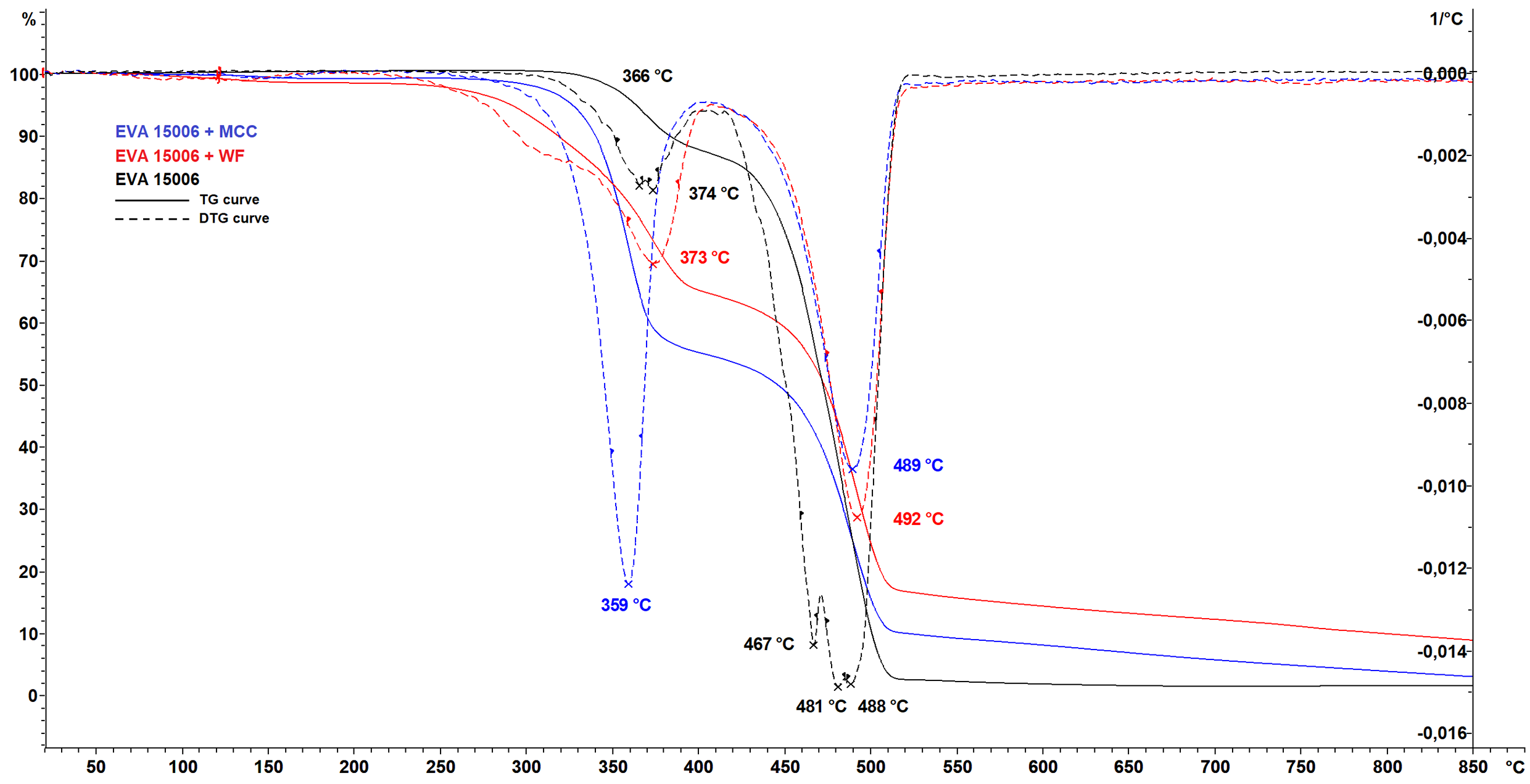

Supplement: Supplementary file 1 [file polymers-16-02103-s001.zip › Figure S1-TGA EVA 15006+Fillers50%.tif]

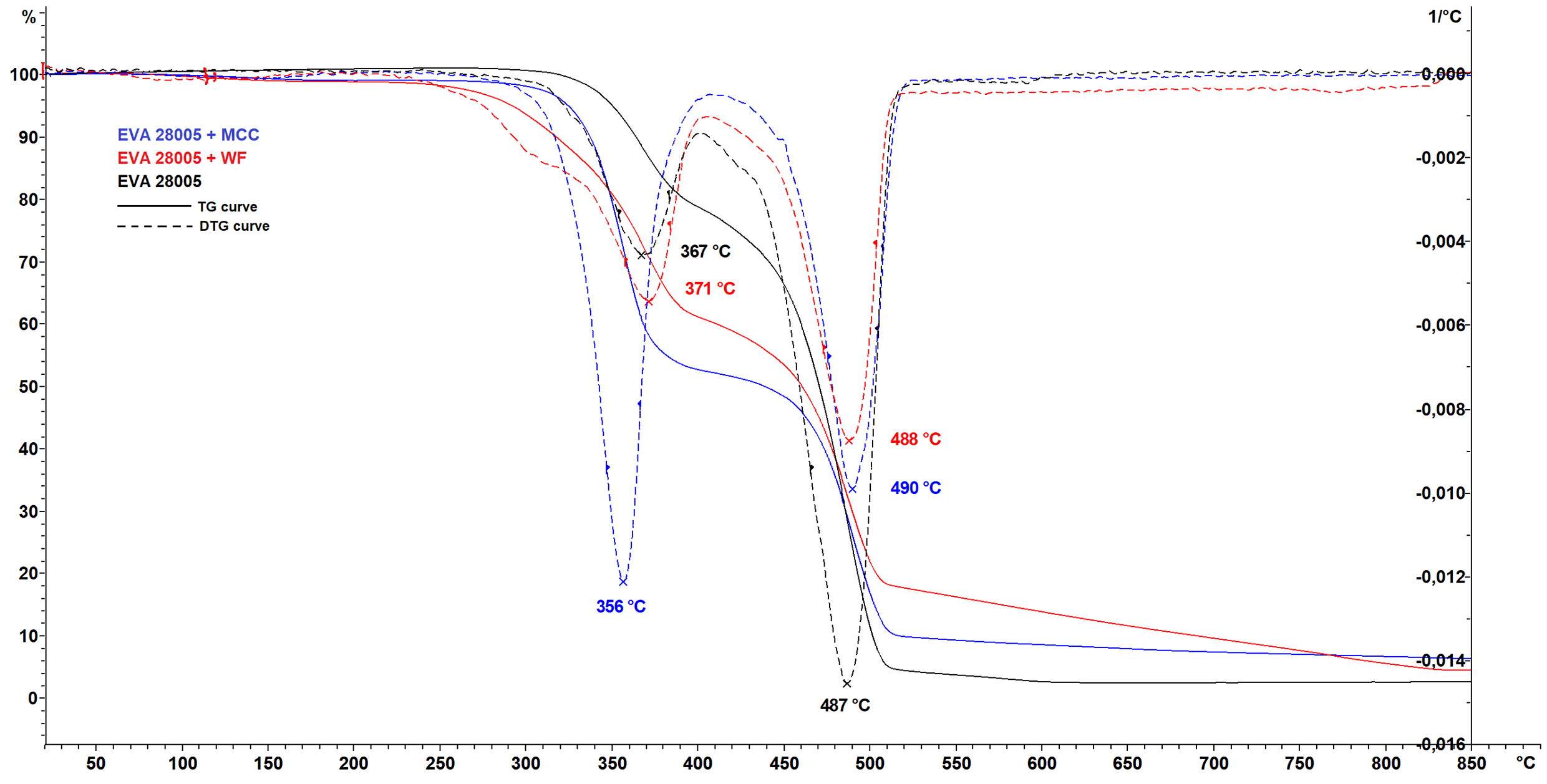

Supplement: Supplementary file 1 [file polymers-16-02103-s001.zip › Figure S2-TGA EVA 28005+Fillers50%.tif]
